# Density-Based Characterization of Microplastics via Cross-Halbach Magnetic Levitation

**DOI:** 10.3390/nano15120941

**Published:** 2025-06-17

**Authors:** Chenxin Lyu, Chengqian Zhang, Baocai Zhang, Xuebin Ni, Hongchao Wang, Peng Zhao

**Affiliations:** 1 The State Key Laboratory of Fluid Power and Mechatronic Systems, College of Mechanical Engineering, Zhejiang University, Hangzhou 310027, China; lyuchenxin@zju.edu.cn (C.L.);; 2 Engineering Research Center of DLIS, Ministry of Education, Hangzhou 310027, China; 3 The Key Laboratory of 3D Printing Process and Equipment of Zhejiang Province, College of Mechanical Engineering, Zhejiang University, Hangzhou 310027, China; 4 Zhejiang Hengdao Technology Co., Ltd., Shaoxing 312051, China

**Keywords:** magnetic levitation, Halbach array, density measurement, characterization of microplastics

## Abstract

The analysis of microplastics poses significant challenges for conventional characterization techniques due to their small size and low concentrations. Magnetic levitation (MagLev), already proven effective for microscale material testing, provides a robust solution for sensitive, accessible, and untethered characterization of such materials. In this paper, we propose a Cross-Halbach magnetic levitation device to measure the densities of microscale plastic materials. Common types of plastic samples, varying in size and concentration, are successfully levitated, and the levitation times are recorded. The samples of common microplastic materials are characterized in less than 180 s. The characterized density values are validated against theoretical results, enabling density-based identification of microplastics. The experimental results demonstrate that the magnetic levitation method is suitable for the characterization of small-sized plastic materials, and the high-speed, low-volume measurement of plastic samples lays the foundation for future applications such as detection, separation, and recycling of ultrafine materials.

## 1. Introduction

Microplastics are typically defined as synthetic polymer particles smaller than 5 mm in diameter [1]. Since their initial identification in marine environments in the early 2000s [2], microplastics have been detected in nearly all environments, including oceans [3], freshwater systems, soil, and even the air [4]. Their persistence and widespread dispersal stem from the fragmentation of larger plastic debris (secondary microplastics) and the direct release of manufactured microparticles (primary microplastics), such as microbeads in personal care products [5] and synthetic textile fibers [6].

The omnipresent microplastics cause tremendous harm and hazard to humans and other life forms. Marine organisms, including plankton, fish, and seabirds, frequently ingest microplastics, leading to physical blockages, reduced nutrient absorption, and chemical toxicity from plastic additives (e.g., phthalates, bisphenol A) or adsorbed pollutants (e.g., PCBs, heavy metals) [7,8]. These particles also enter terrestrial food chains, with recent studies detecting them in agricultural soils [9] and even commercially sold honey [10]. Human exposure pathways include ingestion (via seafood, salt, and drinking water) [11,12] and inhalation of airborne microplastics [4], raising concerns about inflammatory responses and potential carcinogenicity [13].

Given the well-documented environmental and health risks posed by microplastics, research efforts are urgent for effective microplastic pollution management. The precise characterization and profiling of widespread microplastics are crucial for further research, including detection, quantification, and recycling. However, significant methodological challenges hinder the progress in microplastics studies. The extremely small size of many microplastic particles presents substantial analytical difficulties, particularly for conventional optical techniques [14]. Furthermore, the typically low environmental concentrations require highly sensitive detection methods while avoiding false positives from background contamination [15]. Compounding these challenges is the remarkable chemical heterogeneity of environmental microplastics, which undergo complex weathering processes that alter surface properties and chemical signatures [16]. These surface changes, induced by UV radiation, mechanical abrasion, and biological fouling, can significantly interfere with spectroscopic identification methods [17].

Magneto-Archimedes Levitation (MagLev), a technique that levitates diamagnetic samples submerged in paramagnetic solution media [18], is a possible solution to the density-based characterization of microplastics [19,20]. Unlike superconducting levitation, which requires cryogenic conditions and type-II superconductors [21], Magneto-Archimedes Levitation exploits the intrinsic diamagnetism of materials under a magnetic field created by magnets set up in a like-pole-facing pattern, which is usually conducted in a room temperature environment. With density being an innate characteristic of materials, microplastics can be successfully characterized, despite their disadvantages of ultrafine diameter, low concentration, and chemical modification. Furthermore, most microplastics are diamagnetic and insoluble in paramagnetic solutions, making MagLev a possible solution. In macroscale characterization, the magnetic levitation technique has already been widely applied in accurate, sensitive, and untethered testing of a wide range of materials [22,23,24,25], leading to further density-based analysis [26], characterization [27], and separation of different materials [28]. Apart from macroscale testing, magnetic levitation has distinguished itself in microscale density-based analysis and applications due to its unique capability of untethered density characterization regardless of the precise volume of the testing samples. Researchers have successfully applied the magnetic levitation technique to accurate density profiling of microparticles of different sizes [29,30]. Via MagLev device, small objects such as powders [28,29,31,32], droplets [33] or even living materials like cells [34,35,36] were successfully levitated and measured. MagLev has also gained spotlight in biomedical applications, expanding the microscale testing samples from non-living to living materials [37,38].

In this paper, we propose a novel magnetic levitation configuration, namely the Cross-Halbach MagLev. The Cross-Halbach MagLev device is tailor-made for density-based characterization of microplastics, where the small particles stably levitate towards the equilibrium position and form a levitating cluster, whose levitation height can be measured, and the density can be calculated and characterized. We carried out various experiments, including calibration, density characterization of nine common plastics materials on a microscale, and recording levitation times. We also altered the concentrations of materials dispersed in paramagnetic solutions and the sizes of microplastics to confirm the accuracy and reliability of the characterization results. By doing these experiments, we proved that our Cross-Halbach MagLev device is a rapid, accessible, and robust solution for density-based characterization of microplastics.

## 2. Materials and Methods

### 2.1. Design of the Cross-Halbach MagLev Device

**Figure 1:**
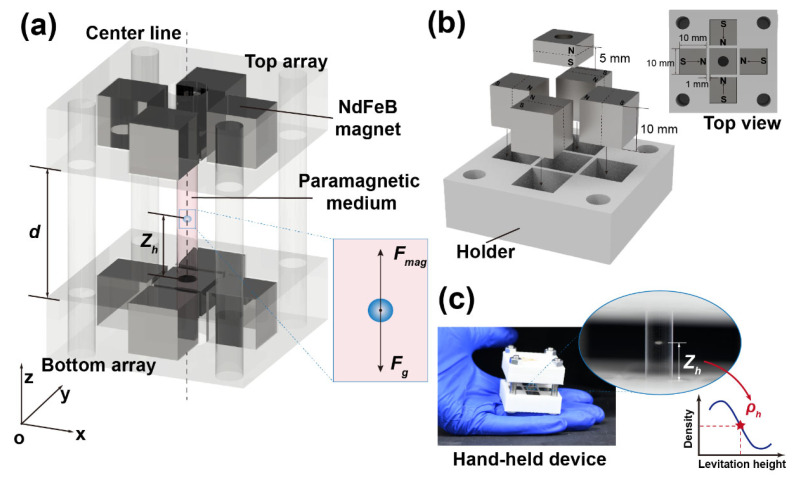
(**a**) The illustration of the proposed Cross-Halbach MagLev device. (**b**) The assembly process of the single Cross-Halbach magnet array. (**c**) The real image of the proposed MagLev device.

The Halbach array is a specialized arrangement of permanent magnets designed to enhance the magnetic field on one side while suppressing it on the opposite side. This configuration is achieved by orienting individual magnets in a spatially rotating magnetization pattern [39]. By putting the two Cross-Halbach arrays in the like-pole-facing pattern, the magnetic field of the Cross-Halbach MagLev device is therefore enhanced. As shown in Figure 1a, the proposed Cross-Halbach MagLev device is designed following basic principles of Magneto-Archimedes Levitation configuration, where two sets of cross magnet arrays were placed in the like-pole-facing fashion along the Z axis, with a certain in-between distance *d*. The testing samples, submerged in paramagnetic medium, are sandwiched between top and bottom arrays and levitate on the center line towards equilibrium position under the coeffect of magnetic force ${\vec{F}}_{mag}$ and the force of gravity ${\vec{F}}_{g}$ (corrected for buoyancy). A cross magnet array consists of five permanent N52 NdFeB magnets, one vertically magnetized (10 × 10 × 5 mm) with a through-hole (diameter of 4 mm) at the center of the cross, and four horizontally magnetized (10 × 10 × 10 mm) surrounding the center. The magnetization directions of the surrounding magnets point to the central magnet, therefore creating a Halbach magnet array in both axis of the cross, where the magnetization vertically rotates by 90°. As illustrated in Figure 1b, the five magnets are fixed in a 3D-printed PLA holder, with 1 mm gap between the central and surrounding magnets for easy assembling. Two identical top and bottom arrays are then connected by four vertically placed bolts, with a distance of 10 mm between the two arrays and the N poles of two central magnets align and facing. A glass test tube filled with microplastic samples dispersed in paramagnetic medium is loaded into the device through the hole on the central magnets. Figure 1c shows the real image of the portable Cross-Halbach MagLev device. Once the test tube is loaded, the dispersed particles start levitating towards the equilibrium position, forming a levitating particle cluster. The levitation height Z_h_ of the cluster is recorded by a digital camera, and according to the correlation between levitation height and density, the testing samples can therefore be characterized.

As illustrated in Figure 1a, the testing sample tend to levitate in the equilibrium position under the effect of magnetic force ${\vec{F}}_{mag}$ and the force of gravity ${\vec{F}}_{g}$ (corrected for the effect of buoyancy) on the centerline along Z axis, as expressed by Equations (1)–(4) [18].

(1) $${\vec{F}}_{\mathrm{mag}}=\frac{\left(\chi_{s}-\chi_{m}\right)}{\mu_{0}}V\left(\vec{B}\cdot \nabla \right)\vec{B}$$

(2) $${\vec{F}}_{g}=\left(\rho_{s}-\rho_{m}\right)V\vec{g}$$

(3) $${\vec{F}}_{g}+{\vec{F}}_{\mathrm{mag}}=\left(\rho_{s}-\rho_{m}\right)V\vec{g}+\frac{\left(\chi_{s}-\chi_{m}\right)}{\mu_{0}}V\left(\vec{B}\cdot \nabla \right)\vec{B}=0$$

(4) $$\rho_{s}={\;\rho }_{m}+\frac{\chi_{s}-\chi_{m}}{{g\mu }_{0}}B_{z}\frac{\partial B_{z}}{\partial z}$$

where $\chi_{m}$, $\rho_{m}$ are the magnetic susceptibility and density of the paramagnetic medium, and $\chi_{s}$, $\rho_{s}$ are of the sample. The magnetic permeability of the vacuum is μ_0_ = 4π × 10^−7^ N/A^2^, V is the volume of the sample, g is the acceleration of gravity. B is the magnetic field in the equilibrium position of the levitated samples. It can be seen that the levitation height of the testing sample depends on the density, regardless of its volume, which makes untethered testing of microplastics possible by reducing the redundant process of volume measurement. According to the theoretical analysis, the relationships between levitation heights and densities can be therefore calculated.

### 2.2. Distribution of Equilibrium Position in Cross-Halbach MagLev

**Figure 2:**
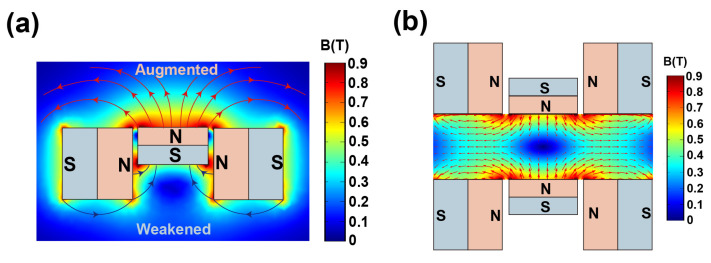
(**a**) Distribution of the magnetic field of the single Cross-Halbach magnet array. (**b**) Distribution of the magnetic field of the proposed Cross-Halbach MagLev device.

To verify the principle of the Halbach array, the magnetic field generated by a single Cross-Halbach array is simulated and illustrated. The simulation model of the Cross-Halbach array was built via COMSOL^®^, where the distributions of magnetic fields of the single array and the MagLev device were calculated and illustrated in Figure 2. Figure 2a shows the distribution of the magnetic field on the central cross-section of the array. It confirms that the principle of the Halbach array still holds for the proposed Cross-Halbach array, where on one side the magnetic field is augmented, while the opposite side is weakened [40]. It is worth noting that the difference in height between the central magnet and the surrounding magnets does not affect this principle. The distribution of the magnetic field of the whole Cross-Halbach MagLev device is illustrated in Figure 2b, which shows the inhomogeneous magnetic field generated by the Cross-Halbach MagLev configuration, where the testing samples, levitating in the paramagnetic solution, tend to move towards the position where the magnetic field is at the minimum [18].

### 2.3. Methods for Density Measuring Process

**Figure 3:**
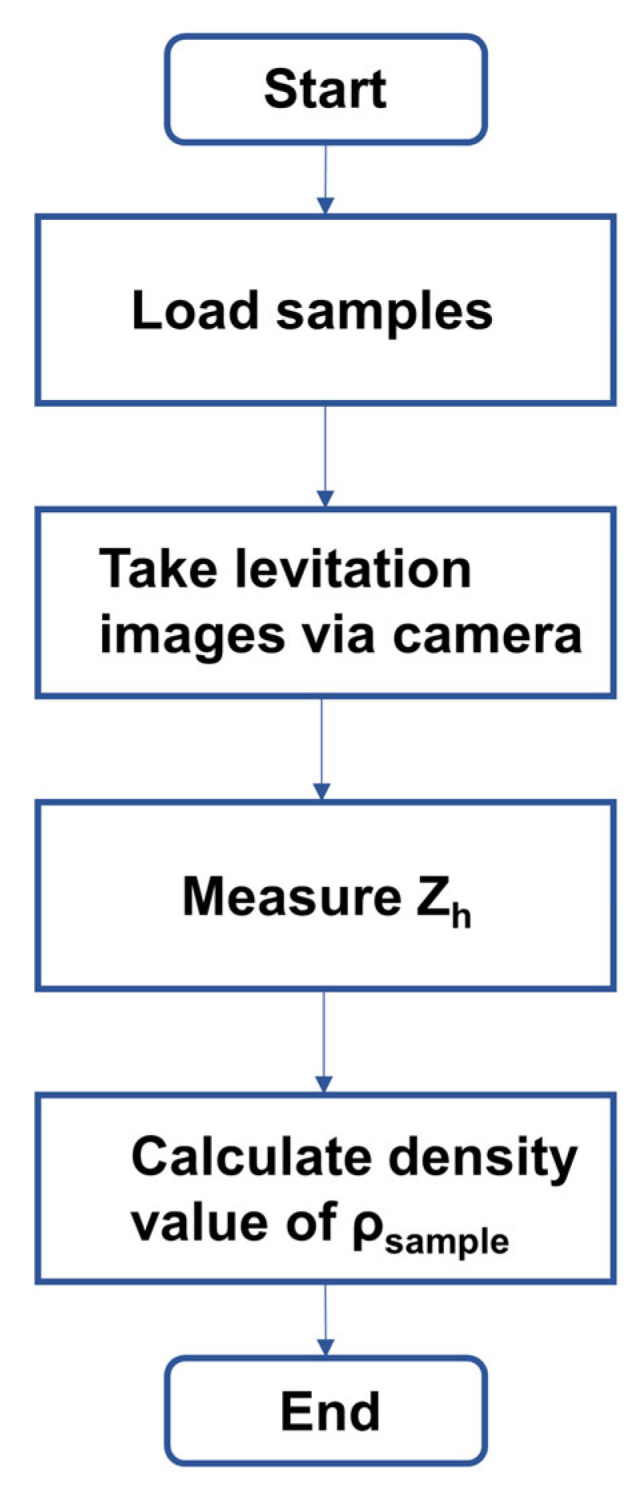
Flow Chart of density-based characterization process.

For density measurement experiments, aqueous solutions in various concentrations of manganese chloride (MnCl_2_·4H_2_O, purchased from Aladdin^®^) are chosen as the paramagnetic medium according to the general density range of common microplastics. The magnetic susceptibility and density of the MnCl_2_ solutions applied in this paper are listed in Table 1. The flow chart of the density-based characterization process is depicted in Figure 3. When the dispersed microplastics reach the equilibrium position and form a visible cluster, with no moving scattered particles, we conclude that the levitation is complete, and then the levitation image is recorded by a digital Nikon^®^ D850 camera. The levitation height, Z_h_, is defined as the distance between the bottom of the container and the geometric center of the cluster. Z_h_ is calculated by counting pixels in the levitation image, where the distance d can also be measured as the number of pixels (N_d_) and levitation height can be measured as the number of pixels (Ns). The levitation height (Z_h_) can be calculated by Equation (5) [22].

(5) $$Z_{h}=\frac{N_{s}}{N_{d}}d$$

## 3. Results and Discussion

### 3.1. Verification and Calibration of the MagLev Device

**Figure 4:**
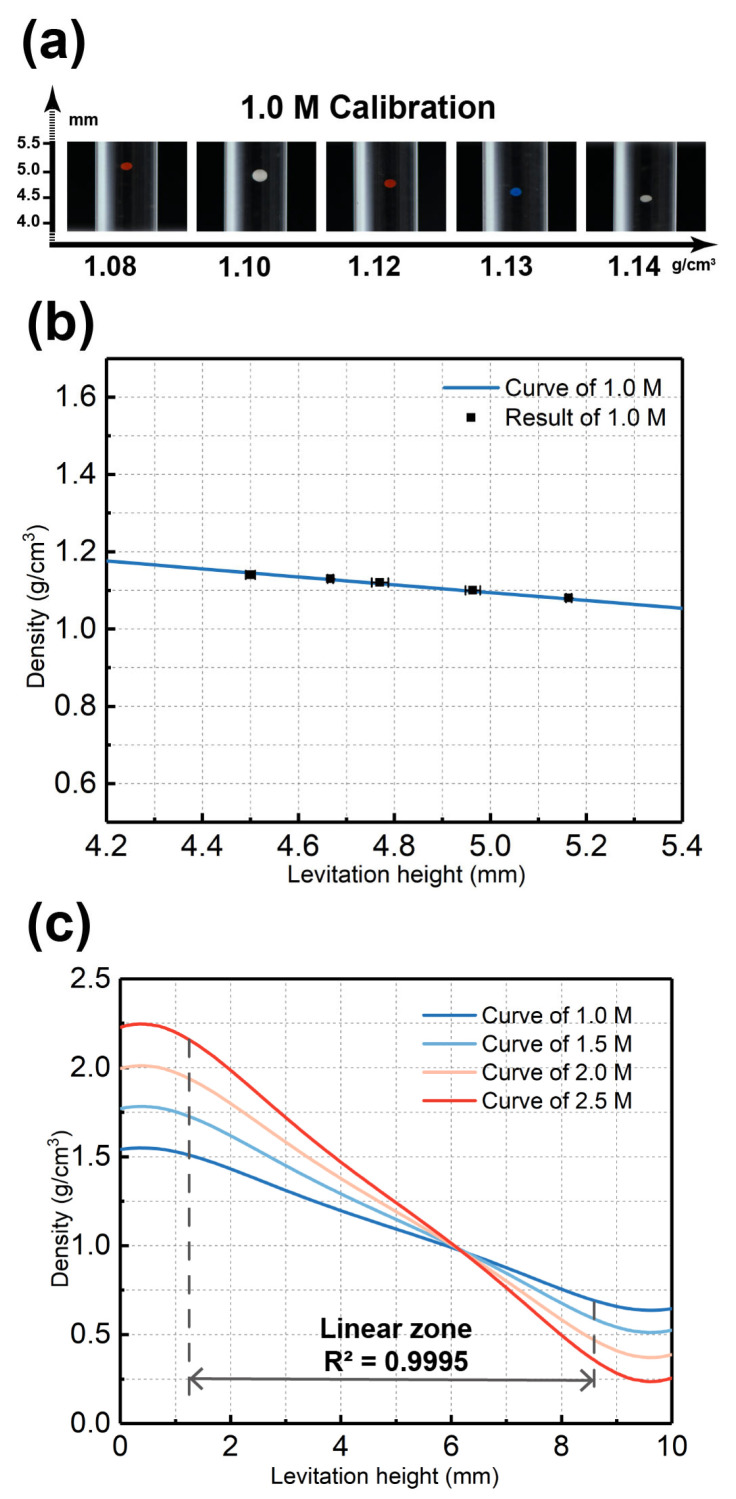
(**a**) Images taken during the calibration experiments with d = 10 mm, where standard beads of 1.08, 1.10, 1.12, 1.13, and 1.14 g/cm^3^ were levitated in 1.0 M MnCl_2_ solution. (**b**) The calibration results of 1.0 M MnCl_2_. (**c**) The relationship between levitation height and density values for 1.0, 1.5, 2.0, and 2.5 M MnCl_2_.

To verify the MagLev theory and calibrate the proposed Cross-Halbach MagLev device, levitation experiments using polyethylene beads with standard density and MnCl_2_ solutions in different concentrations were carried out. The distance d of the Cross-Halbach MagLev was set as 10 mm, and beads of 1.08 (diameter = 425–500 μm, bright red), 1.10 (diameter = 600–710 μm, white), 1.12 (diameter = 500–600 μm, dark red), 1.13 (diameter = 425–500 μm, blue) and 1.14 (diameter = 425–500 μm, white) g/cm^3^ were chosen as standard density beads. For easy observation and measurement, only one bead was injected into the glass container during each levitation and measurement. Before levitation, the bead sank to the bottom of the container. After the glass container was loaded into the MagLev device, the bead moved upwards and levitated towards the equilibrium position, where it stopped in several seconds. The calibration experiment results of the five standard beads levitating in 1.0 M MnCl_2_ solution are shown in Figure 4a. The levitation heights were recorded, as Z_h_ (1.08) = 5.16 mm, Z_h_ (1.10) = 4.95 mm, Z_h_ (1.12) = 4.78 mm, Z_h_ (1.13) = 4.66 mm, and Z_h_ (1.14) = 4.51 mm. The calibration results successfully confirmed the MagLev theory, where all the beads levitate along the centerline, with the heaviest bead (1.14 g/cm^3^, white) levitates at the lowest height and the lightest bead (1.08 g/cm^3^, bright red) levitates at the highest, as can be seen from Figure 4a. The experimental results of the density measurement of standard beads are marked in Figure 4b, where the experimental results match with the calculated curves. The experimental results confirm the relationship between density and levitation height, therefore verifying the accuracy of the proposed Cross-Halbach MagLev device for density characterization of microscale plastics.

### 3.2. Density Characterization of Microplastics

**Figure 5:**
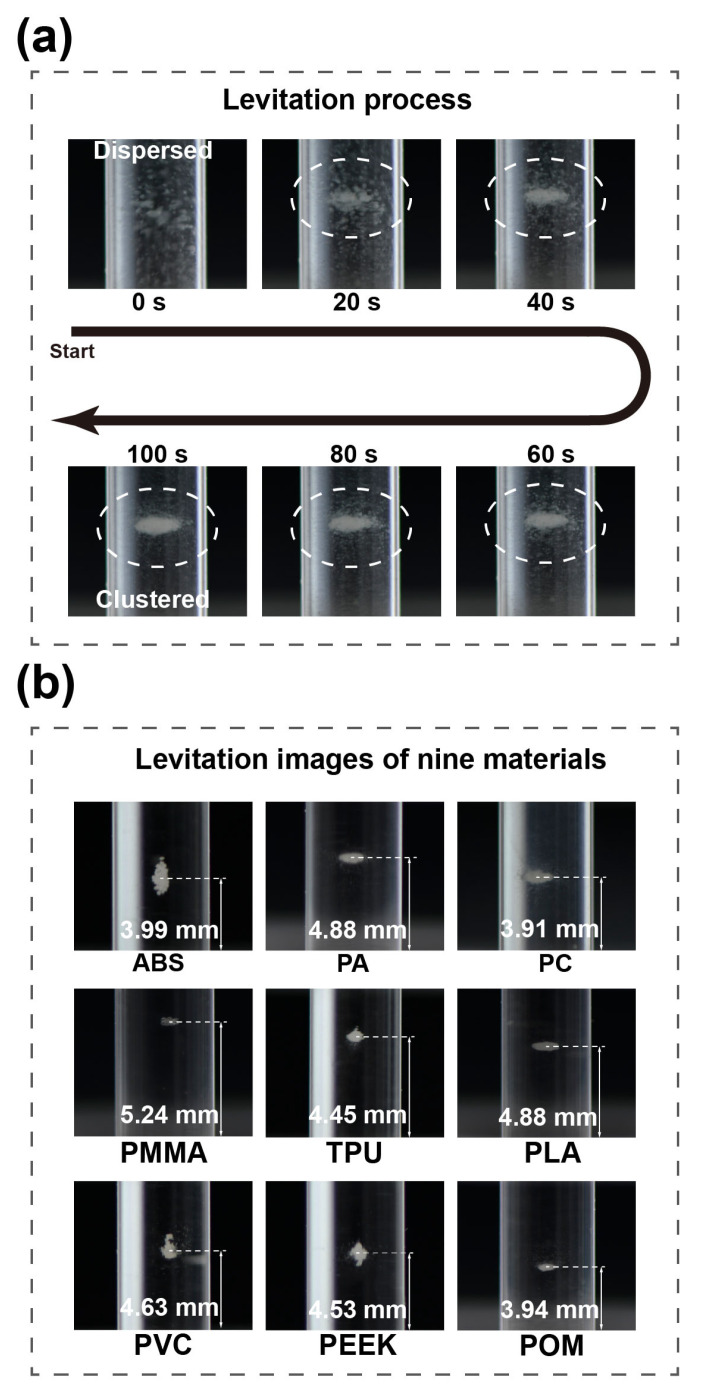
(**a**) The levitation process of PLA particles in 2.5 M MnCl_2_ solution. The formation of the levitating cluster is marked inside the white dashed circle (**b**) The levitation images of nine tested materials.

After calibration, we applied the proposed Cross-Halbach MagLev device to measure the density of common microplastics. The distance d was still set as 10 mm. Here, we chose nine common spherical polymer materials in microscale, with diameter of 150 μm, including acrylonitrile butadiene styrene (ABS), polyamide (PA), polyamide (PC), polymethyl methacrylate (PMMA), thermoplastic polyurethane (TPU), polylactide (PLA), polyvinyl chloride (PVC), polyether ether ketone (PEEK), and polyoxymethylene (POM). These nine materials were separately levitated in different concentrations of MnCl_2_ according to their reference densities, and the concentration of materials dispersed in MnCl_2_ solutions was set as 1.0 mg/mL. The levitation process is shown in Figure 5a, where PLA in 2.5 M MnCl_2_ was levitated. As can be seen in Figure 5a, when levitation started, the microscale particles of PLA were fully dispersed in the container. After the container was loaded into the MagLev device, the microparticles started to levitate from the fully dispersed state. After 20 s of levitation, it can be seen that a small number of particles had reached the equilibrium position and formed a cluster as marked inside the white dashed circle, with other particles still in dispersed state but moving towards the formed cluster. After 40 s of levitation, more particles had reached equilibrium position, and the cluster had grown more compact. As the levitation process went on, the particles kept moving towards the formed cluster, and the levitated particle samples gradually turned from a dispersed state into a clustered state [41,42]. All nine materials can be successfully levitated along the centerline and follow a dispersed-to-clustered process during levitation. The levitation images of these nine materials are shown in Figure 5b. Among these materials, PA, PMMA, TPU, PC, and ABS were levitated in 1.5 M MnCl_2_ solutions, PVC in 2.0 M, and PLA, POM, and PEEK in 2.5 M. The levitation heights of nine polymer clusters were measured separately, where ABS levitated at 3.99 mm, PA at 4.88 mm, PC at 3.91 mm, PMMA at 5.24 mm, TPU at 4.45 mm, PLA at 4.88 mm, PVC at 4.63 mm, PEEK at 4.53 mm, and POM at 3.94 mm. It is worth noting that in some of the levitation images shown in Figure 5b, several scattered particles away from the cluster can be found. This is due to the inhomogeneity of the tested materials.

**Figure 6:**
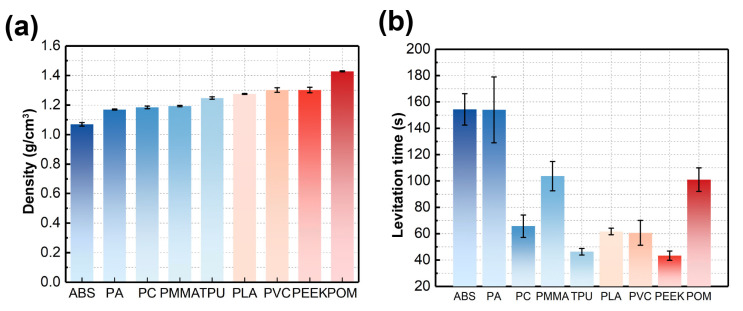
(**a**) Density measurement results of the nine microplastic materials. (**b**) Levitation times of the nine microplastic materials.

The levitation experiments were repeated three times, with images taken and levitation heights recorded and analyzed. We summarized all measurement results and calculated the densities of nine measured materials. The full experimental results are depicted in Figure 6a, where the density profiles of the nine materials were characterized. The density measurement results are listed in Table 2. As the results suggest, the measurement method of magnetic levitation can successfully and accurately measure the densities of common plastics on a microscale.

Levitation time is another factor to consider when applying magnetic levitation. Apart from recording levitation heights and calculating density values, we timed each levitation process of the levitation experiments. The levitation time is recorded as the observation of the levitation process through a digital camera. The time measurement starts when the container is loaded into the MagLev device, where the particles are fully dispersed in the solution, as shown in Figure 5a, and ends when no visible movement of dispersed particles is observed. The levitation times of the measured nine materials are concluded in Figure 6b. It can be seen from the experimental results that different materials have different average levitation times, with PEEK and TPU being the fastest (average time of 43.3 s and 46.2 s, respectively), and ABS and PA being the slowest (average time of 154.3 s and 154.0 s, respectively). According to the results, all nine materials reach a clustered state in a relatively short time span of under 180 s. This may be due to two possible reasons, one being of relatively small levitation volume of the materials and paramagnetic medium, another being of the size effect of magnetic levitation configuration, where the $\left(\vec{B}\cdot \nabla \right)\vec{B}$ is scaled up when the size of the magnetics is scaled down, leading to greater magnetic forces and causing faster levitation speed [36]. The density measurement and levitation timing result prove that the proposed Cross-Halbach MagLev device is capable of measuring common polymer materials in microscales, with a relatively short levitation time and low volume of sample required.

### 3.3. Density Measurement of Altering Concentrations

**Figure 7:**
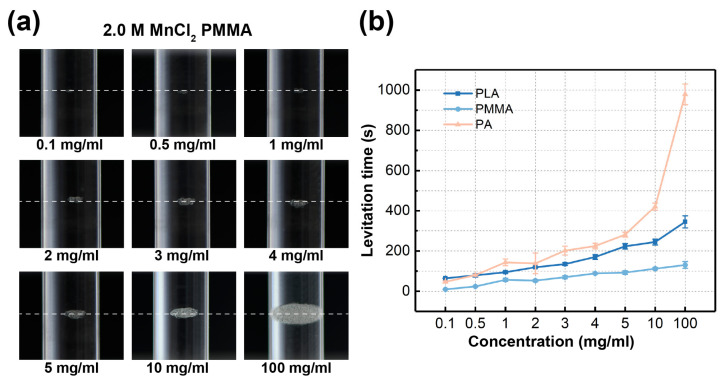
(**a**) The levitation images of PMMA in different concentrations. (**b**) The levitation times of PLA, PMMA, and PA in different concentrations.

The aforementioned experiments prove that the Cross-Halbach MagLev device is capable of measuring densities of common polymer materials regardless of their volume, therefore the densities of microplastics can be precisely measured. Next, we shift our main focus from measurement of density values to levitation time, for which we carried out experiments with altered parameters of the tested materials, including concentrations and sizes of the microplastics. First, we changed the concentrations of materials from 1.0 mg/mL to a range of 0.1, 0.5, 1, 2, 3, 4, 5, 10, and 100 mg/mL. PMMA, PLA, and PA were chosen as testing samples for this experiment. As shown in Figure 7a, PMMA in nine different concentrations, all dispersed in 2.0 M MnCl_2_, were levitated respectively. PMMA in nine concentrations levitated at the same height, as marked by white dashed lines in Figure 7a, confirming that the concentration of microplastics in paramagnetic medium does not affect the levitation height of the materials, therefore it is irrelevant to the measured density. However, the volumes of the formed levitating clusters of the nine concentrations are distinctly different, with the lowest concentration of 0.1 mg/mL only containing two particles, and the highest 100 mg/mL being a large cluster as wide as the glass container. The experimental results prove that the proposed Cross-Halbach MagLev device is capable of levitating microscale materials from single-particle level, to mass analysis of microparticles in more dense concentrations.

The PLA and PA materials were also levitated in the same concentration range, while they were levitated in 2.5 and 1.5 M MnCl_2_ solutions respectively. The time of each levitation experiment was recorded, and the full results are shown in Figure 7b. It can be seen that the three chosen materials show similar trend in levitation time, as the average levitation time increases along with the concentrations. It is because the higher concentration contains more particles, which takes longer to be fully levitated towards the equilibrium positions; therefore, prolongs the average levitation time. These experiments confirm that the alteration of concentrations of materials in paramagnetic medium is irrelevant to the levitation height and measured density, and materials in higher concentrations take a longer time to reach the equilibrium position and form the levitating cluster.

### 3.4. Density Measurement of Altering Particle Diameters

**Figure 8:**
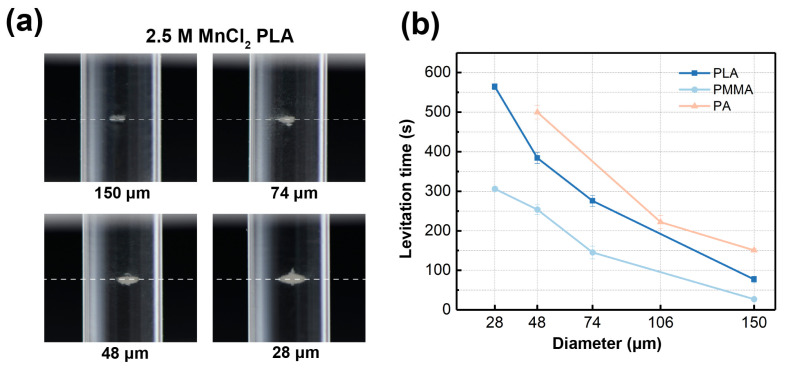
(**a**) The Levitation images of PLA in different diameters. (**b**) The levitation times of PLA, PMMA, and PA in different diameters.

Next, we carried out experiments where we changed the sizes of micromaterials and recorded the levitation time. PLA, PMMA, and PA were still the chosen testing materials. The levitation images of PLA in different sizes are shown in Figure 8a as examples. As can be seen from Figure 8a, PLA with diameters of 150, 74, 48, and 28 μm were separately levitated in 2.5 M MnCl_2_. Same as the concentration experiment, PLA in four different diameters was levitated at the same height, as marked by white dashed lines in Figure 8a. This proves that the size of the material does not affect the measured density value, further confirming the accurate density measurement ability of the proposed Cross-Halbach MagLev device.

The PMMA with a diameter of 150, 74, 48, and 28 μm, and the PA with a diameter of 150, 106, and 48 μm were also levitated in 2.0 and 1.5 M MnCl_2_ solutions, respectively. Still, the time of each levitation experiment was recorded, as shown in Figure 8b. The experimental results suggest that the three chosen materials show the same trend in levitation time, where the levitation time is prolonged while the diameter of the material decreases. This is because particles smaller in size are under the effect of Brownian motion, resulting in longer levitation times [43]. These experiments suggest that the diameter of materials is irrelevant to the levitation height, and particles that are smaller in size take a longer time to reach the equilibrium position than larger particles.

## 4. Conclusions

In summary, we proposed a magnetic levitation configuration tailor-made for the density measurement of plastics on a microscale, namely the Cross-Halbach MagLev device. Via theoretical analysis, simulations, and calibration experiments, we verified the applicability of this MagLev device for precise characterization of densities of microscale polymer particles. Nine common plastic materials in microscale were successfully levitated, with their density values calculated and levitation times recorded. Then, we carried out experiments where we altered the concentrations of microplastics in paramagnetic solutions and the diameters of the particles. The experimental results suggest that the concentration and diameter are irrelevant to the measured density values, further demonstrating the advantage of precise and untethered testing of the proposed MagLev device. The experimental results indicate that the highly accessible and portable Cross-Halbach MagLev device is well-suited for characterizing small plastic materials. Its high-speed, low-volume density measurement capability enables potential future applications, including the detection, separation, and recycling of ultrafine materials.

## Figures and Tables

**Table 1:** Magnetic susceptibility and density of the paramagnetic solutions.

**Concentration of MnCl_2_ (mol/L)**	1.5	2.0	2.5	3.0
$\chi_{m}$ **× 10^−4^ (cm^3^/mol)**	2.77	3.79	4.74	5.49
$\rho_{m}$ **(g/cm^3^)**	1.148	1.192	1.242	1.281

**Table 2:** Density characterization results of different materials.

Material	Medium	Density Value (g/cm^3^)	
		Magnetic Levitation	Pycnometer
ABS	1.5 M MnCl_2_ solution	1.069 ± 0.012	1.069 ± 0.005
PA	1.5 M MnCl_2_ solution	1.169 ± 0.002	1.168 ± 0.002
PC	1.5 M MnCl_2_ solution	1.183 ± 0.008	1.183 ± 0.005
PMMA	1.5 M MnCl_2_ solution	1.192 ± 0.005	1.190 ± 0.005
TPU	1.5 M MnCl_2_ solution	1.247 ± 0.008	1.248 ± 0.003
PLA	2.5 M MnCl_2_ solution	1.275 ± 0.002	1.275 ± 0.003
PVC	2.0 M MnCl_2_ solution	1.301 ± 0.015	1.303 ± 0.005
PEEK	2.5 M MnCl_2_ solution	1.301 ± 0.018	1.301 ± 0.004
POM	2.5 M MnCl_2_ solution	1.428 ± 0.003	1.427 ± 0.005

## Data Availability

Data are contained within the article.
